# Nitrogen Deficiency and Synergism between Continuous Light and Root Ammonium Supply Modulate Distinct but Overlapping Patterns of Phytohormone Composition in Xylem Sap of Tomato Plants

**DOI:** 10.3390/plants10030573

**Published:** 2021-03-18

**Authors:** Martina Paponov, Aleksandr Arakelyan, Petre I. Dobrev, Michel J. Verheul, Ivan A. Paponov

**Affiliations:** 1 NIBIO, Norwegian Institute of Bioeconomy Research (NIBIO), Division of Food Production and Society, P.O. Box 115, NO 1431 Ås, Norway; martina.paponov@outlook.com (M.P.); michel.verheul@nibio.no (M.J.V.); 2 Department of Agronomy, Armenian National Agrarian University, Yerevan 0009, Armenia; arakelyan.alexander@mail.ru; 3 Institute of Experimental Botany, Czech Academy of Sciences, Rozvojova 263, 16502 Prague, Czech Republic; dobrev@ueb.cas.cz; 4 Department of Food Science, Aarhus University, 8200 Aarhus, Denmark

**Keywords:** tomato, nitrogen, nitrate, ammonium, phytohormones, abscisic acid, salicylic acid, jasmonic acid

## Abstract

Continuous light (CL) or a predominant nitrogen supply as ammonium (NH_4_^+^) can induce leaf chlorosis and inhibit plant growth. The similarity in injuries caused by CL and NH_4_^+^ suggests involvement of overlapping mechanisms in plant responses to these conditions; however, these mechanisms are poorly understood. We addressed this topic by conducting full factorial experiments with tomato plants to investigate the effects of NO_3_^−^ or NH_4_^+^ supply under diurnal light (DL) or CL. We used plants at ages of 26 and 15 days after sowing to initiate the treatments, and we modulated the intensity of the stress induced by CL and an exclusive NH_4_^+^ supply from mild to strong. Under DL, we also studied the effect of nitrogen (N) deficiency and mixed application of NO_3_^−^ and NH_4_^+^. Under strong stress, CL and exclusive NH_4_^+^ supply synergistically inhibited plant growth and reduced chlorophyll content. Under mild stress, when no synergetic effect between CL and NH_4_^+^ was apparent on plant growth and chlorophyll content, we found a synergetic effect of CL and NH_4_^+^ on the accumulation of several plant stress hormones, with an especially strong effect for jasmonic acid (JA) and 1-aminocyclopropane-1-carboxylic acid (ACC), the immediate precursor of ethylene, in xylem sap. This modulation of the hormonal composition suggests a potential role for these plant hormones in plant growth responses to the combined application of CL and NH_4_^+^. No synergetic effect was observed between CL and NH_4_^+^ for the accumulation of soluble carbohydrates or of mineral ions, indicating that these plant traits are less sensitive than the modulation of hormonal composition in xylem sap to the combined CL and NH_4_^+^ application. Under diurnal light, NH_4_^+^ did not affect the hormonal composition of xylem sap; however, N deficiency strongly increased the concentrations of phaseic acid (PA), JA, and salicylic acid (SA), indicating that decreased N concentration rather than the presence of NO_3_^−^ or NH_4_^+^ in the nutrient solution drives the hormone composition of the xylem sap. In conclusion, N deficiency or a combined application of CL and NH_4_^+^ induced the accumulation of JA in xylem sap. This accumulation, in combination with other plant hormones, defines the specific plant response to stress conditions.

## 1. Introduction

Continuous light (CL) and a predominant NH_4_^+^ supply are considered prospective treatments to increase crop yield. CL can maximize the daily light integral (DLI), defined as the photosynthetically active light received within a 24 h window, at the same light intensity of an installed lighting setup. The increased DLI directly contributes to enhanced plant growth; however, at the same DLI, plants exposed to CL and lower intensities of photosynthetically active radiation (PAR) grow more quickly than plants grown under periodic light with higher PAR [1,2]. Similarly, plants supplied with NH_4_^+^ can, in theory, show positive growth responses because NH_4_^+^ nutrition is more energetically favorable than nitrate nutrition. Nitrate assimilation in plants initially involves a reduction of NO_3_^−^ to nitrite (NO_2_^−^) by nitrate reductase, followed by reduction of NO_2_^−^ to NH_4_^+^ by nitrite reductase [3], and these steps are highly energy demanding [4]. Supplying N as NH_4_^+^ bypass this high demand for reductant required for conversion of NO_3_^−^ to NH_4_^+^.

Despite these theoretical considerations of the positive effects of CL and NH_4_^+^ on plant growth, these factors, in fact, usually have negative effects in many plant species, including tomato [5,6]. CL induces leaf chlorosis, down-regulates photosynthesis, stimulates the accumulation of carbohydrates, and accelerates leaf senescence [5,7]. Interestingly, an exclusive NH_4_^+^ supply can induce partially overlapping symptoms to those seen with CL treatment; specifically, it can induce chlorosis, decrease chlorophyll concentration, and increase the accumulation of non-structural carbohydrates [8]. The similarity between the symptoms induced by CL and by NH_4_^+^ supply might indicate an involvement of overlapping or common mechanisms of plant responses to these environmental stimuli.

One way to confirm the existence of a close cross-talk between mechanisms in response to different factors is to investigate the interaction of these factors in a full factorial experiment. The simplest assumption holds that factors act independently if their combined application results in an additive effect, whereas synergetic or antagonistic effects indicate the involvement of close cross-talk between mechanisms regulating the plant response to the factors. Synergetic or antagonistic interactions describe the situation wherein the output of the combined application of factors is higher or lower, respectively than the sum of the outputs of these factors when applied alone. The interaction between CL and NH_4_^+^ has remained essentially uninvestigated and requires more study.

The potential benefits of using CL and NH_4_^+^ for crop production prompted the present investigation into the physiological mechanisms behind plant responses to CL and NH_4_^+^ supply because this knowledge forms the basis for targeted breeding. Investigation of plant responses to CL has confirmed the important role of type III light-harvesting chlorophyll a/b binding protein 13 (CAB-13) in plant resistance to CL. CAB-13 functions by balancing energy transfer between photosystem I and II during the light-harvesting reactions [9]. However, linking CL-tolerance to the expression of CAB-13 does not reveal a clear mechanism for the CL response [10]. Further analysis of possible mechanisms involved in CL responses has suggested that circadian asynchrony seems to be the main factor inducing the observed CL-induced injury [10].

Interestingly, as with CL, the potential targets for responses to a predominant NH_4_^+^ supply are also the photosystems, with the D1 protein of PSII playing a role [11]. However, several other hypotheses have implicated other mechanisms to explain the NH_4_^+^-sensitivity of plants. These mechanisms include essential ion imbalances, such as decreases in K^+^, Ca^2+^, Mg^2+^, and increases in SO_4_^2−^ and/or PO_4_^3−^, disruptions in the pH gradients across plant membranes, uncoupling of photophosphorylation, futile and energy-intensive transmembrane cycling of the NH_4_^+^ ion, oxidative stress, and disturbance of the N-glycosylation of proteins [6,12].

In addition to these previously proposed mechanisms, plant hormonal signaling might also be involved in plant responses to both CL and NH_4_^+^. A contribution of CL to the modulation of hormonal signaling has been demonstrated in Arabidopsis seedlings, where CL stabilized 1-aminocyclopropane-1carboxylic acid (ACC) synthase 7 (ACS7) protein, the rate-limiting enzyme in ethylene biosynthesis [13]. In potato shoot cultures, significant differences were observed between the diurnal light (DL) and CL conditions in the levels of indole auxins, cytokinin ribosides, salicylic acid (SA), abscisic acid (ABA), and phaseic acid (PA) [14]. Experiments with altered light–dark periods also indicated a role for cytokinins because a much higher sensitivity to prolonged light periods is observed in cytokinin-deficient mutants than in wild-type plants [15].

Different forms of N can also activate different hormonal signaling pathways and make plants more efficient at N uptake and utilization [16]. For example, exposure to NO_3_^−^ can trigger local, long-distance, and systemic signaling to coordinate NO_3_^−^ uptake, NO_3_^−^ utilization, and plant growth under conditions of varying levels of N availability or of unequal distribution of NO_3_^−^ ions in the soil [17,18,19]. The key role of NO_3_^−^ ion signaling is related to the long-distance root-to-shoot transport of plant cytokinin hormones, as a 24 h replacement of NO_3_^−^ with NH_4_^+^ in experiments with tobacco reduced the leaf growth rate without reducing the N concentration in the leaves and correlated with a reduction of active cytokinin levels [20]. In addition to regulating leaf growth, cytokinins play roles in the regulation of lateral root development in NO_3_^−^-rich soil zones. NO_3_^−^ regulates cytokinin synthesis through the activity of IPT genes (IPT3, IPT5, and IPT7) that are necessary for increases in lateral root development in NO_3_^−^-rich zones in response to long-distance N depletion [21].

Nitrate modulates cytokinin levels, but it also regulates ABA signaling. Evidence for this comes from studies on *Commelina communis*, which responds to NO_3_^−^ application to the roots by increasing the stomatal response to ABA [22]. This effect seems to be related to the modulation of the pH of the xylem sap. Specifically, increased availability of NO_3_^−^ to the roots causes a significant alkalinization of the xylem sap. This pH change, in turn, enhances the accumulation of ABA as a weak acid in the apoplast, according to the anion-trap concept [23], and the higher pH increases the proportion of the charged form of ABA, thereby reducing the diffusion of ABA through the plasma membrane.

A predominant NH_4_^+^ supply can also directly modulate hormonal signaling and contribute to additional plant responses that also appear to involve ABA [24]. Indeed, NH_4_^+^ exposure caused a 3-fold enhancement of the root-to-shoot transport of ABA [25]. One possible molecular link between NH_4_^+^ and ABA is the plastid metalloprotease AMOS1/EGY [26]. Transcriptome analysis has shown that, among the genes activated in response to NH_4_^+^, 90% were regulated by AMOS1/EGY1. Moreover, a majority of the AMOS1/EGY1-dependent and NH_4_^+^-activated genes carry a core motif of ABA-responsive elements in their promoters. Thus, NH_4_^+^ most likely triggers a chloroplast retrograde signal initiated by reactive oxygen species (ROS) and leads to leaf chlorosis, whereas the AMOS1/EGY1–dependent response recruits the ABA signaling pathway to protect leaves from chloroplast damage [26]. Hence, under NH_4_^+^ stress, activated retrograde signaling by NH_4_^+^ recruits downstream ABA signaling to regulate the expression of NH_4_^+^-responsive genes in the nucleus and thereby prevents NH_4_^+^ toxicity.

The similarity of the injury symptoms induced by CL and a predominant NH_4_^+^ supply, coupled with the finding that light conditions and the form of N supply can both modulate hormonal signaling, led us to hypothesize that hormonal signaling components might be involved in overlapping mechanisms responsible for plant responses to these environmental stimuli. Whether the interaction between CL and NH_4_^+^ occurs, and whether plant hormones are involved in this interaction, are not yet known. Here, we have addressed the possibility of an interaction between CL and the form of supplied N by conducting a full factorial experiment with two light conditions (DL and CL) and two forms of N (NO_3_^−^ vs. NH_4_^+^). We have evaluated the effect of these factors on plant growth, the carbohydrate and ion composition of shoots, and the hormone composition of xylem sap. We further investigated the role of the form of supplied N under DL by comparing plants supplied exclusively with NO_3_^−^ or NH_4_^+^ with plants grown under N-deficiency or with a combined application of both N forms.

## 2. Materials and Methods

Tomato seeds (*Solanum lycopersicum* Mill cv. Ailsa Craig and cv. Rio Grande) were sterilized in 2.5% sodium hypochlorite solution for 15 min and then washed thoroughly 5 times with deionized water. The sterilized tomato seeds were germinated in “sandwich” filter paper placed between mat layers (Class Ohlsen) and fixed in plastic plates. Seeds were sown in a line 3 mm below the top of the filter paper leaving a distance of 7–10 mm between seeds. Seeds were incubated in a 20% nutrient solution containing 500 µM KNO_3_. For the first 2 days, the seeds were kept in darkness at 18 °C.

### 2.1. Experiment with Strong Stress

Seedlings (cv. Ailsa Craig and cv. Rio Grande) were transferred at day 12 after sowing (DAS) from the “sandwich” system to 2.2 L plastic pots containing 50% nutrient solution and 0.2 mM KNO_3_ or 0.1 mM (NH_4_)_2_SO_4_ under diurnal light with 16 h/8 h day/night photoperiod, 22 °C/18 °C day/night temperature, and 77%/90% day/night air humidity. The intensity of the light provided by Heliospectra LED RX30 (Gothenburg, Sweden) lights (with nine diode channels: 370, 400, 420, 450, 530, 630, 660, 735, and 5700 K white light) was adjusted to 280 µmol m^−2^ s^−1^ PAR. Four seedlings per pot (2 pots per treatment) were fixed with foam slabs onto the lid and further treated as needed with either NO_3_^−^ (KNO_3_) or NH_4_^+^ (NH_4_)_2_SO_4_) to prevent depletion of N from the nutrient solution. N concentrations were ranging from 0.2–0.5 mM. After plant adaption under these conditions for 3 days (15 DAS), one half of plants was maintained by cultivation in diurnal light and the other half was transported into the continuous light chamber and 22 °C temperature. All seedlings were further cultivated at one plant per pot in the full nutrient solution, which contained 1 mM CaSO_4_, 1 mM K_2_HPO_4_, 1 mM KH_2_PO_4_, 2 mM MgSO_4_ [27], and micronutrients with the following concentrations: 15 µmol Fe, 10 µmol Mn, 5 µmol Zn, 30 µmol B, 0.75 µmol Cu, and 0.5 µmol Mo. Every treatment was represented by 4 pots. The pH of the nutrient solution was monitored regularly and controlled between pH 5.5 and 6.3. The nutrient solution was replaced every 5 days. The nutrient solution was continuously aerated. Plants were sampled at 26 DAS and 27 DAS in the continuous and periodic light conditions, respectively. Ultimately, plants were exposed for 14 or 15 days to different forms of N and for 11 or 12 days to continuous and diurnal light conditions, respectively.

### 2.2. Experiment with Mild Stress

Seedlings (cv. Ailsa Craig) were transferred at 10 DAS to a 0.8 L pot containing 50% nutrient solution and 1 mM KNO_3_. At 26 DAS, the seedlings were transferred to pots containing 2.4 L full nutrient solution with the following four N treatments: N deficiency (no nitrogen), NO_3_^−^ (KNO_3_) alone, NH_4_^+^ ((NH_4_)_2_SO_4_) alone, and an equimolar mixture of NH_4_^+^ and NO_3_^−^. Before and after transfer, these plants were grown under a 12 h day/12 h night photoperiod (diurnal light condition) with the same light source, day/night temperature, and air humidity as in the experiment with strong stress. Nitrogen was applied every day based on the N demand of the plants to avoid depletion of nitrogen from the nutrient solution. Depending on the size of the plants, the concentration of N in the nutrient solution varied from 200 µM to 1 mM. At 29 DAS, another portion of the seedlings was transferred to NO_3_^−^ or NH_4_^+^ supply alone and the CL conditions. Plants were sampled at 36 DAS (diurnal light) and 39 DAS (continuous light). Ultimately, plants were exposed for 10 days to the different forms of N and different light conditions.

### 2.3. Measurements of Plant Growth

After the treatments, four or six plants per treatment were sampled. The fresh weights of leaves, stems, and roots were measured separately. Images of the leaves were captured with a NIKON d750 camera. The individual leaf areas and total leaf area were estimated using ImageJ software. Leaves, stems, and roots were dried for 2 days at 65 °C and weighed. In the mild stress experiment, we sampled separately 3 distal leaflets of leaf 3 (cotyledons were not counted), these were weighed, photographed and immediately frozen in liquid nitrogen and stored at −80 °C until further mineral ion, nonstructural carbohydrate, N content and pigment analysis.

### 2.4. One-Step Extraction of Chlorophyll, Anions, Cations and Sugars

Metabolites were extracted from leaves and separated using a two-phase separation method described by Salem [28]. The samples of the 3 leaflets were lyophilized for approx. 48 h BK-FD10S (BIOBASE, Shandong, China) and the leaf material was powdered (Star-Beater with 5 mm metal balls, 29 Hz for 3 min). A 20 mg sample of powder was weighed into a 2 mL Eppendorf microcentrifuge tube and extracted following the extraction described by Salem [28].

In brief, 1 mL of a pre-cooled (−20 °C) methyl tert-butyl ether MTBE:MeOH (3:1 vol/vol) solution was added per sample immediately vortexed until the tissue was well homogenized (1–2 min) and then incubated on a shaker at 100 rpm for 45 min at 0–5 °C, followed by an ice-cold sonication bath (USC300TH) for 15 min. For phase separation, 650 µL of H_2_O:MeOH (3:1 vol/vol) was added and vortexed thoroughly for 1–2 min. After centrifuging (Micro Star 17) at 17,000× *g* at 4 °C for 15 min, the nonpolar phase and the (semi)polar phase were separated. The aliquots of these phases and the insoluble pellet were frozen and stored at −20 °C. In total, we obtained a volume of 750 µL nonpolar MTBE extract and 900 µL (semi)polar water:methanol extract.

### 2.5. Quantitative Determination of Pigments

The chlorophyll *a*, (Chl *a*), Chl *b*, total chlorophyll (Chl *a+b*) and total carotenoid contents were quantitatively determined spectrophotometrically from the upper (semi)polar phase Salem [28]. We measured pigment concentration, using the method and equations developed by [29]. The pigments were measured immediately after extraction in a fume hood by diluting the upper polar phase 20 times with 100% methanol (1:20 vol/vol) and pipetting 3 technical replicates of 200 µl volume into a 96 well plate. The absorption was measured by UV-VIS spectrophotometry (Multiskan™ FC Microplate Photometer, Thermo Scientific™, Waltham, MA, USA) at 470 nm, 652 nm and 665 nm with three technical replications; the results for triplicate measurements were averaged before statistical analysis. A mixture of MTBE:methanol. (1:20, vol/vol) was taken as blank. Measurements were made quickly to avoid evaporation. Prior to using the Lichtenthaler equations, we modified the absorption values according to [30] to allow direct translation of Lichtenthaler’s equation, developed for use with 1 cm path length spectrophotometers, to the microplate. The blank values were subtracted from absorption values. The path length of the microplate was determined as (microplate absorbance—microplate blank)/(spectrophotometer absorbance—blank). For the absorbance of a 200 µl sample volume leaf extract in MTBE:methanol (1:20), we used the path length correction factor of 0.51, which corresponds to the value obtained by Warren et al. 2007 for the path length for the same volume of pure methanol. The Lichtentaler equations were then used to calculate the pigment concentrations.

### 2.6. Chlorophyll Index

The chlorophyll index was measured with a Hansatech Instruments Chlorophyll Content System (CL-01, Norfolk, UK) on at least 2 leaves (leaf 2 and leaf 3). Every leaf was measured three times and the average values were used for statistical analysis.

### 2.7. Analysis of Nonstructural Carbohydrates from the Water:Methanol Phase

Glucose, fructose, and sucrose in the (semi)polar phase (see Section 2.4) were quantified using a sequential enzymatic assay in microplates [31]. The (semi)polar solvent phase was diluted 1:8 with 80% ethanol and triplicate 20 µL aliquots of each sample were pipetted into separate wells of a 96-well UV-Star microplate (Greiner). The microplate, without standards added, was placed into an oven at 50 °C to dry for 60 min.

For the calibration curve, 20 µL of a standard glucose solution (0, 0.005, 0.0125, 0.025, 0.050, 0.125, 0.25, to 0.5 mg mL^−1^ in DI water, prepared weekly) was added in triplicate to each microplate and assayed for glucose, fructose, and sucrose according to [31] using a glucose assay reagent (G3293, Supelco) according to the manufacturer’s instructions. The glucose concentration was measured at 340 nm with a Multiskan™ FC Microplate Photometer. Glucose, fructose, and sucrose absorption values were calculated based on triplicate replications and used in statistical analysis, as suggested by [31].

### 2.8. Extraction of Starch

The same enzymatic assay [31] was applied to detect the starch content by measuring glucose released from starch after solubilizing and hydrolyzing the insoluble pellet from the liquid extraction according to Salem [28]. The pellet was first detached from the 2 mL Eppendorf tube and transferred to a 5 mL Eppendorf tube with screw cap, being washed twice with 1.5 mL 80% ethanol and centrifuged at 3000× *g* for 15 min. A second identical washing step with 3 mL 80% ethanol followed, and the supernatant was discarded.

We then followed the general procedures described by Hendrix et al. [32] and [31], but the starch was hydrolyzed with α-amylase (Sigma A 4582) and α-amyloglucosidase from *Aspergillus niger* (11202367001, Roche), according to manufacturer’s instructions. After hydrolysis, tube content was brought to 4 mL with DI water (+/−0.05 g) and mixed, and then the insoluble material was spun down (10 min /3000× *g*) and the supernatant was removed. The supernatant was further diluted 20 times with DI water before assaying for glucose.

### 2.9. Ion Chromatographic Determination of Anions and Cations

The ion composition analysis of leaves was performed by ion chromatography with conductive detection, as described in Paponov, et al. [33]. Prior to analysis, the extracted (semi)polar phase from the one-step extraction was diluted 20-fold and 50-fold with deionized water for cations and anions, respectively, and filtered through 0.2 µm nylon syringe filters (Pall Corporation, Port Washington, NY, USA), discarding at least 1 mL of the first filtrate.

### 2.10. Determination of Total Nitrogen by Alkaline Persulfate Digestion

For total nitrogen determination, we digested the leaves in alkaline persulfate [34] and analyzed the digest by ion chromatography. A 4 mg ± 0.1 sample of the powdered material from the distal part of leaf 3 was suspended in 7.9 mL distilled water in a 15 mL Falcon tube and 0.1 mL of a 50% w/w NaOH and 0.018 g of potassium persulfate (K_2_S_2_O_8_) were added without mixing. The tubes were immediately closed and wrapped in aluminum foil, put into an autoclave bag with distilled water beneath, and then autoclaved at 120 °C for 1.5 h. The samples were allowed to cool down overnight.

The total N content was determined by ion chromatography of the nitrate ion. The digest was diluted 15 × with distilled water and filtered through a 0.45 µm hydrophilic sterile syringe filter and discarding the first 1.2 mL filtrate. The rest of the filtrate was used for anion determination with a Metrosep A Supp 5 column, with an eluent consisting of 3.2 mM Na_2_CO_3_ and 1.0 mM NaHCO_3_ supplied at 1 mL min^−1^ and no acetone, to avoid peak co-elution. The suppression of the background conductivity, and the identification and quantification of the nitrate were as described for ion chromatography determination of anions and cations.

### 2.11. Xylem Sap Collection

Plants were transferred in fresh complete nutrient solutions (pH 5.9, aerated, with the desired N concentration provided by 1 mM KNO_3_, 0.5 mM (NH_4_)_2_SO_4_, 0.5 mM KNO_3_ plus 0.25 mM (NH_4_)_2_SO_4_, or zero N) two hours before decapitation. The xylem sap was sampled by the root pressure method [35]. The cut surface was cleaned with deionized water and a silicon tube was fixed over the stump and sealed with silicone grease. The xylem exudate was collected with a pipette after a 1 h run for a period of 60 min, stored in plastic vials on ice, and subsequently frozen in liquid nitrogen.

### 2.12. Hormone Analysis

A 500 µL volume of xylem sap was filtered through a 10,000 MW cut-off centrifugal filter unit (Amicon Ultra 0.5, Merck, Kenilworth, NJ, USA) according to the supplied user guide. A 200 µl sample of the filtrate was then freeze-dried (BK-FD10S, BIOBASE, Shandong, China). Samples were purified and analyzed using a previously described method [36,37]. The samples were dissolved in cold (−20 °C) methanol/water/formic acid (15/4/1 *v*/*v*/*v*) and the following stable isotope-labelled internal standards (10 pmol/sample) were added: ^13^C_6_-IAA (Cambridge Isotope Laboratories, Tewksbury, MA, USA); ^2^H_4_-SA (Sigma-Aldrich); ^2^H_3_-PA, ^2^H_3_-DPA, ^2^H_4_-7OH-ABA, ^2^H_5_-ABA-GE (NRC-PBI); ^2^H_6_-ABA, ^2^H_2_-OxIAA, ^2^H_5_-JA, ^2^H_5_-transZ, ^2^H_5_-transZR, ^2^H_5_-transZ7G, ^2^H_5_-transZ9G, ^2^H_5_-transZOG, ^2^H_5_-transZROG, ^2^H_5_-transZRMP, ^2^H_3_-DZ, ^2^H_3_-DZR, ^2^H_3_-DZ9G, ^2^H_7_-DZOG, ^2^H_3_-DZRMP, ^2^H_6_-iP, ^2^H_6_-iPR, ^2^H_6_-iP7G, ^2^H_6_-iP9G, and ^2^H_6_-iPRMP (Olchemim, Olomouc, Czech Republic). Solid phase extraction (SPE) of the plant extract on a reverse phase–cation exchange SPE column (Oasis-MCX, Waters, Milford, MA, USA) resulted in two fractions: the acid fraction, eluted with methanol (auxins, ABA, SA, JA) and the basic fraction, eluted with 0.35 M NH_4_OH in 60% methanol (CKs, ACC). Fractions were analyzed by HPLC (Ultimate 3000, Dionex, Sunnyvale, CA, USA) coupled to the 3200 Q TRAP hybrid triple quadrupole/linear ion trap mass spectrometer (Applied Biosystems, Waltham, MA, USA). The hormones were quantified by the isotope dilution method with multilevel calibration curves (r^2^ > 0.99). Data processing was carried out with Analyst 1.5 software (Applied Biosystems, Waltham, MA, USA).

### 2.13. Statistics

Data were statistically analyzed by analysis of variance (two-way and three-way ANOVA). The treatments were replicated four or six times. When significant treatment effects were indicated by ANOVA, Fisher’s protected LSD test was used to compare the individual means (Statistica 13 software package, TIBCO, Palo Alto, CA, USA).

## 3. Results

### 3.1. Interaction of Continuous Light with N Forms on Tomato Plant Growth Traits

#### 3.1.1. Experiment with Strong Stress

**Figure 1:**
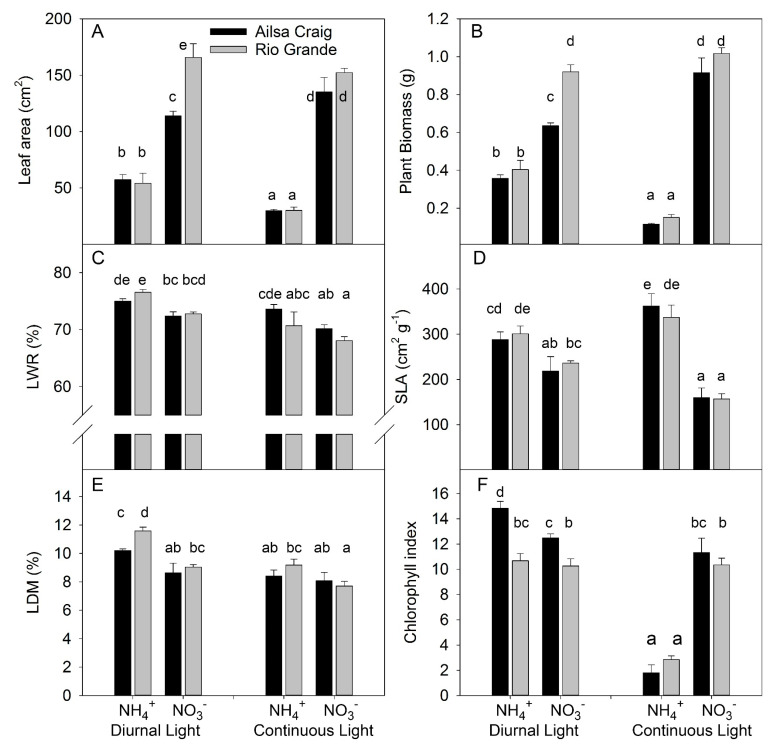
Experiment with strong stress. The effect of light treatment (diurnal or continuous) and N form (NO_3_^−^ or NH_4_^+^) on leaf area (**A**), plant biomass (**B**), leaf weight ratio (LWR) (**C**), specific leaf area (SLA) (**D**), leaf dry matter content (LDM) (**E**), and chlorophyll index (**F**) for tomato genotypes cv. Ailsa Craig or cv. Rio Grande. (*n* = 4). Differences between means with different letters are statistically significant. Tomato seedlings were transferred at day 12 after sowing (DAS) to nutrient solution with NO_3_^−^ or NH_4_^+^ under diurnal light. At 15 DAS, one half of the plants was maintained by cultivation in diurnal light and the other half was exposed to continuous light. Plants were sampled at 26 DAS and 27 DAS in the continuous and diurnal light conditions, respectively. Ultimately, plants were exposed for 14 or 15 days to different forms of N and for 11 or 12 days to continuous and diurnal light, respectively.

Two tomato genotypes (cv. Ailsa Craig and cv. Rio Grande) were grown under diurnal light (DL) or continuous light (CL) in a nutrient solution where N was supplied exclusively as NO_3_^−^ or as NH_4_^+^ at a N concentration ranging from 0.2 to 0.5 mM. Previous investigations showed that these genotypes have contrasting responses to different N forms: cv. Ailsa Craig shows decreased growth under NH_4_^+^ compared to NO_3_^−^-based solution [38], whereas cv. Rio Grande grows better under NH_4_^+^ than NO_3_^−^ supply [8]. We analyzed the effect of light and N form on major plant growth traits of both genotypes by destructive analysis of plants sampled after 11–12 days light treatment and 14–15 days N treatment. Leaf area and growth responded similarly to the investigated treatments, supporting the functional relationship between these traits (Figure 1A,B). The most profound observation was the significant interaction between light and N forms for leaf area and biomass because the simultaneous application of CL and NH_4_^+^ had a synergetic inhibitory effect on leaf area and plant growth (Figure 1A,B, Table S1, Figure S1). This synergetic effect was observed in both genotypes, despite the contrasting response to NH_4_^+^ observed in the previous investigation [8,38]; this similarity indicates that the synergy reflects a common tendency in tomato plants. A significant interaction was also found between genotype and N forms because, under DL conditions, the leaf area and biomass were higher for Rio Grande than for Alisa Craig under NO_3_^−^ supply, whereas no differences between genotypes were found under NH_4_^+^ supply. Under DL, the greater allocation of DM to leaves (LWR) under ammonium supply (Figure 1C) indicates that this trait does not contribute to the reduction in plant growth because the higher allocation of DM to leaves benefits plant growth under non-limiting N supply [39]. CL decreased LWR, which reflects the common dry matter allocation to leaves under higher daily light integrals [40]. The significant interaction between genotype and light (Table S1) indicates different genotypic responses to CL, as LWR was more strongly reduced under CL in Rio Grande than in Ailsa Craig.

Exclusive NH_4_^+^ supply increased the SLA (Figure 1D), showing that plant growth reduction occurred despite a more efficient investment of DM in leaves for leaf area formation. The SLA and leaf dry matter content (LDM) in leaves affect the leaf thickness [41], which is important for photosynthetic rate [42,43]. Under DL, NH_4_^+^ supply decreased leaf thickness due to the increased SLA and higher value of LDM (Figure 1E). However, under CL, NH_4_^+^ decreased leaf thickness, mostly due to an increase in SLA.

Chlorophyll (Chl) concentration, measured as the Chl index showed that this was the most sensitive trait in the light vs. N form interaction. Under DL, NH_4_^+^ increased Chl content for Ailsa Craig but did not change it for Rio Grande. CL combined with NO_3_^−^ supply did not change the Chl content in either genotype. However, CL under NH_4_^+^ supply strongly reduced Chl in both genotypes (Figure 1F, Table S1). The genotypic differences in leaf area and plant biomass under DL and exclusive NO_3_^−^ supply were not related to dry matter allocation to leaves, specific leaf area, and/or the chlorophyll index in the leaves, assuming a higher net assimilation rate for Rio Grande than for Ailsa Craig under this condition.

#### 3.1.2. Experiment with Mild Stress

**Figure 2:**
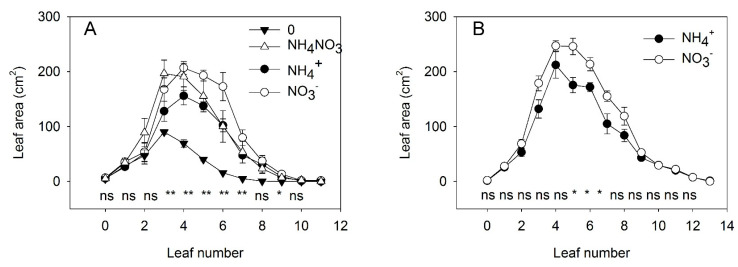
Experiment with mild stress. The effect of N forms and light conditions on the area of individual leaves. The area of individual leaves of tomato plants (cv. Ailsa Craig) 10 days after 4 different N treatments: exclusively NO_3_^−^, exclusively NH_4_^+^, no N supply (“zero”), or a mixed supply of NO_3_^−^ and NH_4_^+^ under diurnal light supply (**A**). The effect of different N forms (NO_3_^−^ or NH_4_^+^) under continuous light (**B**). Nitrogen was supplied as KNO_3_ or (NH_4_)_2_SO_4_ every day to maintain the N concentration in a range between 0.2 and 1 mM (*n* = 4 for diurnal light and *n* = 6 for continuous light). Asterisks indicate significant differences among treatments (* *p* < 0.05 and ** *p* < 0.01), whereas “ns” indicates no significant difference (*p* < 0.05).

In the second experiment, we started the experimental treatments at a later stage of plant development (26 DAS) to reduce the injuries induced by CL and NH_4_^+^ and to investigate the physiological response to these factors when morphological differences were absent or less visible than under strong stress. Because the synergetic effects between CL and NH_4_^+^ on leaf area, plant growth, and Chl concentration were similar for both tomato genotypes, we used only cv. Ailsa Craig in this experiment. Under mild stress conditions, the pattern of reduction in individual leaf area by replacement of NO_3_^−^ with NH_4_^+^ was similar for DL and CL; therefore, no synergetic effect on leaf area was observed between CL and NH_4_^+^. Under DL, supplying NH_4_^+^ alone did not restrict the emergence of new leaves, although it did tend to reduce leaf expansion once the leaves had emerged (Figure 2A). Under CL, NH_4_^+^ alone again did not restrict the emergence of new leaves. Significant differences in leaf area between NO_3_^−^ and NH_4_^+^ nutrition were observed for leaves 5, 6, and 7 from the cotyledons, but the size of the older leaves did not differ between plants grown with NO_3_^−^ and NH_4_^+^ supply under CL (Figure 2B). The early period of leaf development is characterized by extensive cell replication, whereas cell elongation is slow [44]. These findings, therefore, indicate that NH_4_^+^ did not inhibit cell elongation since leaf size was unchanged for older leaves, where the cell number had already been defined before the different treatments were applied.

For deeper insight into the interaction between NO_3_^−^ and NH_4_^+^ under DL, we added a treatment of N deficiency (no N supply) and a supply of a mixture of NH_4_^+^ and NO_3_^−^ (in equal N-proportions). These treatments showed that N deficiency strongly reduced the emergence of new leaves and restricted the expansion of the leaves that had emerged before the transfer of the plants to the N-deficient solution (Figure 2A). Simultaneous application of NO_3_^−^ and NH_4_^+^ did not show any additive positive effect of supplying both forms of N. Specifically, a mixed application of NO_3_^−^ and NH_4_^+^ had the same effect on the size of older leaves as was achieved with NO_3_^−^ as the sole N source; however, the effect on the size of new leaves was similar to that observed when NH_4_^+^ was the sole N source.

**Figure 3:**
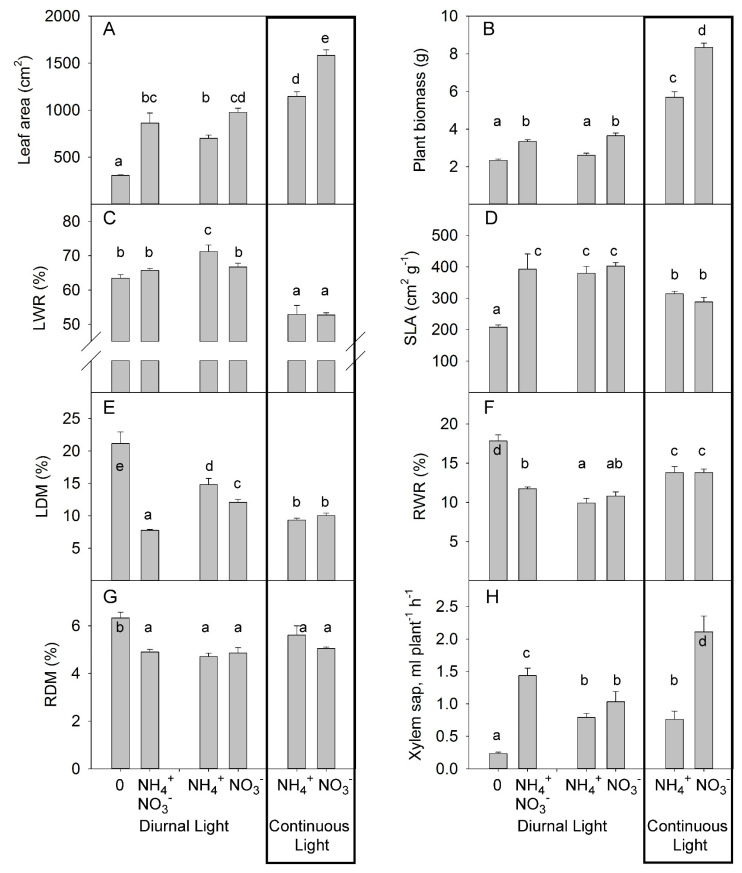
Experiment with mild stress. The effect of different N forms and light conditions on leaf area (**A**), plant biomass (**B**), leaf weight ratio (LWR) (**C**), specific leaf area (SLA) (**D**), leaf dry matter content (LDM) (**E**), root weight ratio (RWR) (**F**), root dry matter content (RDM) (**G**), and the rate of xylem sap flux (**H**) from decapitated wild-type tomato plants cv. Ailsa Craig (*n* = 4 for diurnal light and *n* = 6 for continuous light). Differences between means with different letters are statistically significant. At day 26 after sowing (DAS), seedlings were transferred to either nutrient solution without nitrogen (treatment 0), NO_3_^−^ (KNO_3_) alone, NH_4_^+^ ((NH_4_)_2_SO_4_) alone, or an equimolar mixture of NH_4_^+^ and NO_3_^−^ and diurnal light conditions. At 29 DAS, another portion of the seedlings was transferred to nutrient solution with NO_3_^−^ or NH_4_^+^ and continuous light conditions. Plants were sampled at 36 DAS (diurnal light) and 39 DAS (continuous light). Ultimately, plants were exposed for 10 days to different forms of N and different light conditions.

These results for individual leaf sizes were further supported by a similar reduction in total leaf area of 28% and 27% under DL and CL, respectively, indicating a similar stress intensity induced by NH_4_^+^ supply under either DL or CL (Figure 3A). Interestingly, N deficiency more strongly decreased total leaf area than did the replacement of NO_3_^−^ by NH_4_^+^, indicating that NH_4_^+^ supply partly offset the NO_3_^−^ functions for leaf development. The mixture of NO_3_^−^ and NH_4_^+^ supply under DL did not significantly affect leaf area compared to NO_3_^−^ supply alone (Figure 3A).

We also estimated the effect of light and N forms on growth and major plant growth traits by destructive analysis of plants sampled after 10 days of the different N treatments. The relative reduction of biomass due to replacement of NO_3_^−^ by NH_4_^+^ was 28% for DL and 32% for CL (Figure 3B), indicating that plant growth showed a similar response to the stress induced by replacement of NO_3_^−^ by NH_4_^+^ under both light treatments, with no synergetic interaction between CL and NH_4_^+^. Interestingly, in contrast to the observation for leaf area, the N-deficient plants and NH_4_^+^-grown plants both had similar weights. These measurements showed that the N-deficient plants had a less pronounced reduction in plant biomass than in leaf area in comparison with the NH_4_^+^-grown plants (Figure 3A,B). Simultaneous application of NO_3_^−^ and NH_4_^+^ had similar effects on growth as observed for NO_3_^−^ supply alone, with no additive or inhibitory effects on growth (Figure 3B).

The decrease in dry matter allocation to leaves under CL reflects the common plant response to increased daily light integral [45]; under this condition (CL), no differences were found between NO_3_^−^ and NH_4_^+^ supply (Figure 3C, Table S2). Under DL, NH_4_^+^ enhanced the DM allocation to leaves. N-deficiency and simultaneous application of NO_3_^−^ and NH_4_^+^ did not alter the DM allocation to the leaves beyond that seen in plants supplied exclusively with NO_3_^−^. The allocation of the same percentage of DM to leaves (LWR) by N-deficient and NO_3_^−^-fed plants indicates that this trait does not contribute to the differences observed in plant growth between N-deficient and NO_3_^−^-fed plants.

Replacement of NO_3_^−^ by NH_4_^+^ did not change the SLA, whereas CL reduced the SLA for both NO_3_^−^ and NH_4_^+^ supply (Figure 3D). This reduction occurred despite the lower values of LDM (Figure 3E), indicating that the reduced SLA was not due to an increased LDM but rather to a greater thickness of the leaves. Nitrogen deficiency caused a reduced SLA compared with the other treatments, indicating that SLA is the major trait responsible for the reduction in leaf area in N-deficient plants (Figure 3D). Simultaneous application of NO_3_^−^ and NH_4_^+^ had the same effect on SLA as an application of a single form of N. Thus, under DL, the SLA was mainly regulated by the amount of N available, rather than by the specific form of N. The strong (75%) increase in LDM in N-deficient plants compared to the NO_3_^−^ fed plants indicated that LDM was the main trait responsible for the reduction in SLA (by 48% for N deficient vs. NO_3_^−^ fed plants) (Figure 3E). Interestingly, the combined application of NO_3_^−^ and NH_4_^+^ further reduced the LDM when compared with NO_3_^−^-fed plants.

No differences were noted on dry matter partitioning in roots between NH_4_^+^ and NO_3_^−^ as the predominant N supply (Figure 3F). Plants allocated more DM to the roots when grown under CL than when grown under DL, independent of the form of N supply. Nitrogen deficiency increased DM allocation to the roots (Figure 3F). Simultaneous application of NO_3_^−^ and NH_4_^+^ caused a weak increase in the root weight ratio (RWR) over that seen with NH_4_^+^ supply alone and a tendency for an increase over that seen with NO_3_^−^ supply alone (Figure 3F). The N forms and light treatments did not significantly change the root dry matter content (RDM). N-deficient plants showed the highest RDM (Figure 3G, Table S2).

Under DL, the xylem sap flux from NH_4_^+^-supplied plants was not significantly lower than the flux from plants supplied with NO_3_^−^ (Figure 3H). Under continuous light, replacement of NO_3_^−^ with NH_4_^+^ strongly reduced the rate of xylem sap flux, indicating that the inhibitory effect of NH_4_^+^ supply on xylem sap rate occurs only under CL. N deficiency strongly decreased the rate of xylem sap flux. Simultaneous application of NO_3_^−^ and NH_4_^+^ increased the rate of xylem sap flux in comparison with the applications of single N forms.

### 3.2. Effects of Different N Forms on the Hormonal Composition of Xylem Sap

**Figure 4:**
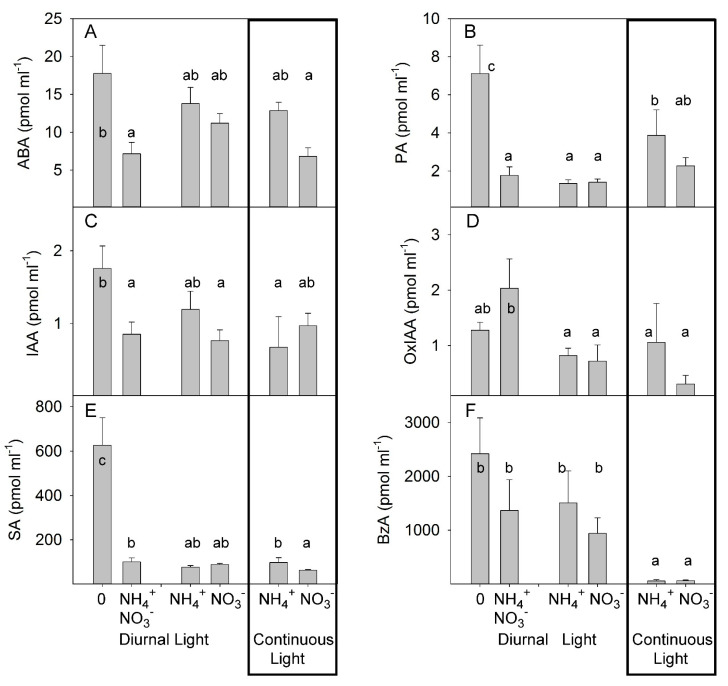
Experiment with mild stress. The effect of different N forms and light conditions on concentrations (pmol ml^−1^) of abscisic acid (ABA) (**A**), phaseic acid (PA) (**B**), indole-3-acetic acid (IAA) (**C**), oxo-indole-3-acetic acid (OxIAA) (**D**), salicylic acid (SA) (**E**), and benzoic acid (BzA) (**F**) in xylem sap from decapitated tomato plants cv. Ailsa Craig (*n* = 4). Differences between means with different letters are statistically significant. At day 26 after sowing (DAS), seedlings were transferred to either nutrient solution without nitrogen (treatment 0), NO_3_^−^ (KNO_3_) alone, NH_4_^+^ ((NH_4_)_2_SO_4_) alone, or an equimolar mixture of NH_4_^+^ and NO_3_^−^ and diurnal light conditions. At 29 DAS, another portion of the seedlings was transferred to nutrient solution with NO_3_^−^ or NH_4_^+^ and continuous light conditions. The xylem sap was sampled by the root pressure method at 36 DAS (diurnal light) and 39 DAS (continuous light).

We examined how different N forms and light conditions affected long-distance hormone transport from root-to-shoot by decapitating tomato plants and collecting the xylem sap that exuded from the root stumps by root pressure for 1 h. Analysis of the hormone concentrations in the collected sap revealed that the xylem sap ABA concentrations were not significantly different in plants grown under NH_4_^+^ or NO_3_^−^ supply alone. Light treatment did not significantly change the ABA concentration in xylem sap. The highest concentrations of ABA tended to occur in the xylem sap of decapitated N-deficient plants, whereas the ABA content was lower in the xylem sap of plants grown under combined–N supply (Figure 4A), further indicating that NH_4_^+^ supply, per se, did not enhance the accumulation of ABA in xylem sap. ABA is catabolized to PA, which is physiologically inactive [46]. Under DL, no differences in PA concentration were found between NH_4_^+^ and NO_3_^−^ treatments. CL tended to increase the PA concentration in NO_3_^−^-grown plants, but the highest impact of CL was observed in the NH_4_^+^-grown plants, supporting a synergetic effect between CL and NH_4_^+^ on the accumulation of PA. The xylem sap level of PA was also strongly increased in N-deficient plants compared with other treatments of plants under DL (Figure 4B, Table S3). This indicated a higher conversion of ABA to PA and/or an inhibition of PA utilization in N-deficient plants.

The effect of different forms of N and light conditions on auxin homeostasis was relatively weak, as only a moderate increase in IAA concentration was detected in N-deficient plants. By contrast, an increase was noted in oxo-IAA (OxIAA) concentration when both N forms were simultaneously applied, suggesting only a minor role for auxin in long-distance root-to-shoot signaling (Figure 4C,D). SA levels showed a similar pattern to that of PA and showed no differences under DL between NO_3_^−^- and NH_4_^+^. Under continuous light, the SA concentration was higher in NH_4_^+^-grown plants than in NO_3_^−^-grown plants. The strongest increase in SA concentration was seen in N-deficient plants (Figure 4E). Under DL, the levels of benzoic acid (BzA), the precursor of SA, were not significantly different between NO_3_^−^- and NH_4_^+^-grown plants, whereas CL strongly reduced the BzA concentration. N deficiency caused no significant modulation of BzA concentration (Figure 4F), indicating that the regulation of SA accumulation under this condition was due to a higher rate of conversion of BzA to SA than of BzA biosynthesis in N-deficient plants.

**Figure 5:**
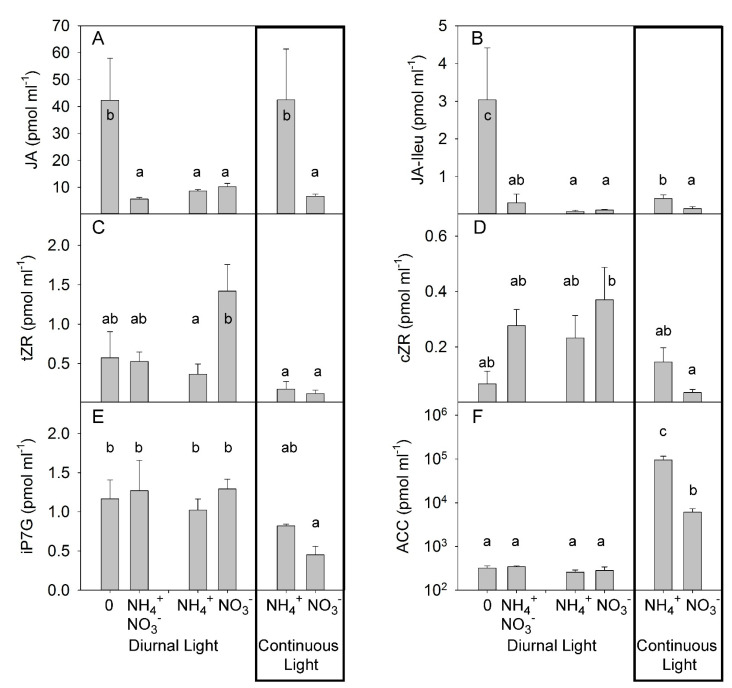
Experiment with mild stress. The effect of different N forms and light conditions on concentrations (pmolml-1) of jasmonic acid (JA) (**A**), JA-isoleucine (JA-Ileu) (**B**), trans-zeatin riboside (tZR) (**C**), cis-zeatin riboside (cZR) (**D**), isopentenyl adenine-7-glucoside (iP7G) (**E**), and 1-aminocyclopropane-1-carboxylic acid (ACC) (**F**) in xylem sap from decapitated tomato plants cv. Ailsa Craig (*n* = 4). Differences between means with different letters are statistically significant. At day 26 after sowing (DAS), seedlings were transferred to either nutrient solution without nitrogen (treatment 0), NO_3_^−^ (KNO_3_) alone, NH_4_^+^ ((NH_4_)_2_SO_4_) alone, or an equimolar mixture of NH_4_^+^ and NO_3_^−^ and diurnal light conditions. At 29 DAS, another portion of the seedlings was transferred to nutrient solution with NO_3_^−^ or NH_4_^+^ and continuous light conditions. The xylem sap was sampled by the root pressure method at 36 DAS (diurnal light) and 39 DAS (continuous light).

The other hormone compounds known to play important roles under biotic stresses, namely jasmonic acid (JA) and JA-Ileu, responded similarly to PA (Figure 5A,B). Under DL, JA and JA-Ileu concentrations did not differ significantly in response to different N forms (Figure 5A,B). This observation was consistent with the findings reported in a previous publication, where NH_4_^+^ supply did not change the level of JA in citrus plants [47]. However, under CL, JA and JA-Ileu concentrations were significantly higher in the NH_4_^+^-supplied plants than in NO_3_^−^-grown plants, showing the synergetic effect of CL and NH_4_^+^ on JA concentrations in xylem sap (Figure 5A,B, Table S4). Under DL, N-deprivation strongly increased the concentration of JA and JA-Ileu in the xylem sap of N-deficient plants, and combined application of NO_3_^−^ and NH_4_^+^ yielded a similar JA accumulation to that seen with the application of single N forms. Under DL, the concentration of the cytokinin tZR was higher in NO_3_^−^-grown plants than in NH_4_^+^-grown plants (Figure 5C). No effect of N form was found for two other cytokinins: cZR and iP7G (Figure 5D,E). CL significantly reduced the concentration of all measured cytokinins N deficiency and application of the mixture of NO_3_^−^ and NH_4_^+^ did not significantly change the cytokinin concentration when compared with the single NO_3_^−^ and NH_4_^+^ treatments. (Figure 5C–E). Under DL, different forms of N supply did not significantly modulate the concentration of the ethylene precursor 1-aminocyclopropane-1-carboxylic acid (ACC) in xylem sap. CL strongly increased the accumulation of ACC with the highest effect observed in combination with NH_4_^+^ supply (Figure 5F, Table S4). Under DL, N deficiency or combined application of NO_3_^−^ and NH_4_^+^ did not change the concentration of ACC over that seen following the application of N as a single form.

### 3.3. Interaction between N Forms and Light on Total Nitrogen, Leaf Pigment, Non-Structural Carbohydrate, and Mineral Ion Concentrations in Leaves

**Figure 6:**
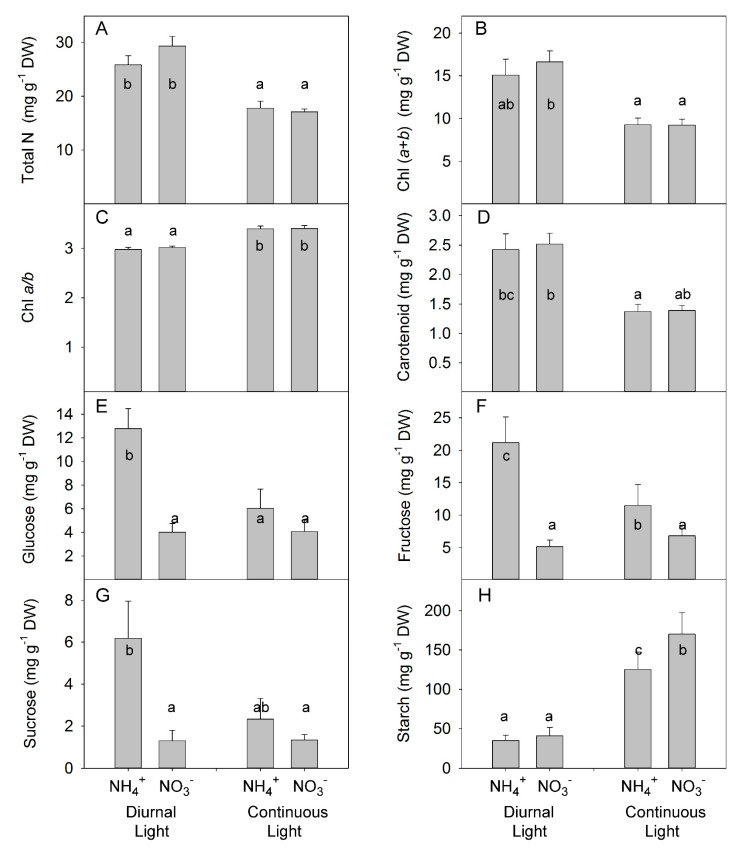
Experiment with mild stress. The effect of different N forms and light conditions on total nitrogen (N) (**A**), chlorophyll (**B**,**C**), carotenoid (**D**), glucose (**E**), fructose (**F**), sucrose (**G**), and starch (**H**) concentrations in leaves of tomato plants cv. Ailsa Craig. Differences between means with different letters are statistically significant. At day 26 after sowing (DAS), seedlings that had been growing under diurnal light conditions were transferred to nutrient solution with NO_3_^−^ (KNO_3_) alone or NH_4_^+^ ((NH_4_)_2_SO_4_) alone. At 29 DAS, another portion of the seedlings was transferred to nutrient solution with NO_3_^−^ or NH_4_^+^ and continuous light conditions. Plants were sampled at 36 DAS (diurnal light) and 39 DAS (continuous light). Ultimately, plants were exposed for 10 days to different forms of N and different light conditions.

N forms did not change the total concentration of N in leaves; however, CL strongly reduce the concentration of total N (Figure 6A, Table S5), in agreement with a previous observation of the effect of CL on N concentration in leaves [48]. The lower total N concentration in leaves in CL-grown plants was related to a lower Chl concentration, in agreement with the assumption that a higher daily light integral requires less light-capturing machinery [49]. Under both light conditions, no differences were found between N forms, indicating that the intensity of NH_4_^+^ stress was not sufficient to induce the typically observed stress symptoms (such as leaf chlorosis) (Figure 6B, Table S5). Similarly, the Chl *a*: Chl *b* ratio was lower under DL than under CL (Figure 6C), which is in agreement with the common plant response of a reduction in this ratio that increases the light-harvesting complex under low light availability [49]. Other pigments, like carotenoids, can extend the spectral range over which light drives photosynthesis and can protect against harmful photodynamic reactions the carotenoid levels were reduced under CL, indicating that the DL-form leaves are more capable of efficient light absorption and expanding the spectral range (Figure 6D).

A significant interaction between N form and light treatment was found for soluble sugars (i.e., glucose, fructose, sucrose) (Table S5). Under DL, NH_4_^+^ supply increased accumulation of all soluble sugars; however, under CL, no differences were found between NH_4_^+^ and NO_3_^−^ (Figure 6E–G). By contrast, under DL, NH_4_^+^ supply did not change starch accumulation; however, under CL, NH_4_^+^ increased starch accumulation (Figure 6H).

**Figure 7:**
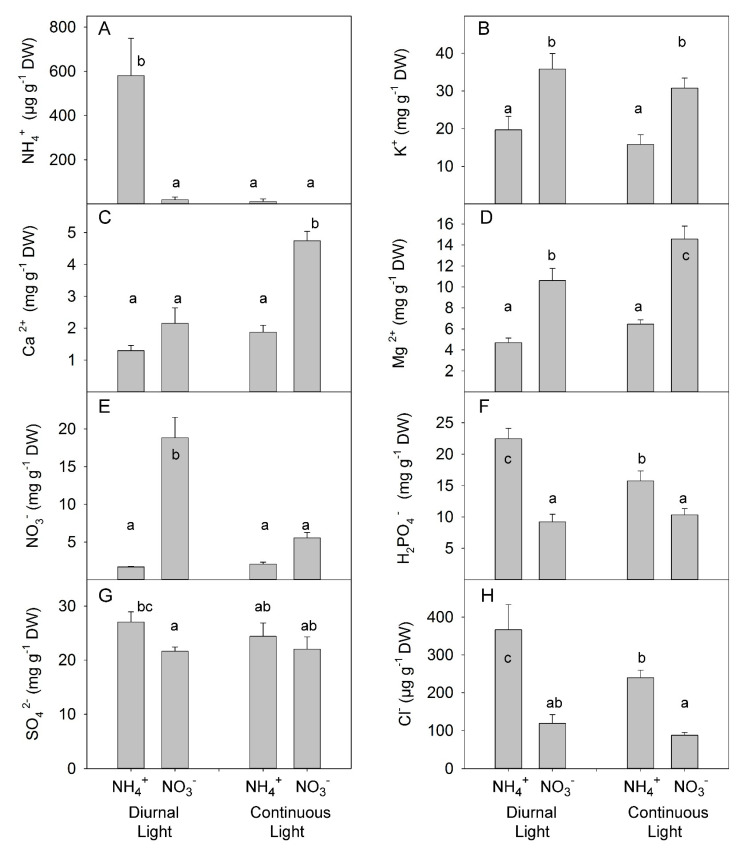
Experiment with mild stress. The effect of different N forms and light conditions on ammonium (**A**), potassium (**B**), calcium (**C**), magnesium (**D**), nitrate (**E**), dihydrogen phosphate (**F**), sulfate (**G**), and chlorine (**H**) concentrations in leaves of tomato plants cv. Ailsa Craig. Differences between means with different letters are statistically significant. At day 26 after sowing (DAS), seedlings that had been growing under diurnal light conditions were transferred to nutrient solution with NO_3_^−^ (KNO_3_) alone or NH_4_^+^ ((NH_4_)_2_SO_4_) alone. At 29 DAS, another portion of the seedlings was transferred to nutrient solution with NO_3_^−^ or NH_4_^+^ and continuous light conditions. Plants were sampled at 36 DAS (diurnal light) and 39 DAS (continuous light). Ultimately, plants were exposed for 10 days to different forms of N and different light conditions.

Analysis of ion concentration in leaves showed the antagonistic effect of simultaneous application of CL and NH_4_^+^ on ammonium accumulation: NH_4_^+^ accumulated under DL; however, no differences were found between NH_4_^+^ and NO_3_^−^ treatments under CL (Figure 7A, Table S6). As expected, the replacement of NO_3_^−^ with NH_4_^+^ reduced the accumulation of K^+^ [50]. Light treatment did not change the concentration of K^+^ in leaves (Figure 7B). Under DL, N forms did not change the concentration of Ca^2+^ in the leaves; however, under continuous light, NO_3_^−^-treated plants accumulated about 2-fold higher concentration than NH_4_^+^-grown plants (Figure 7C). NO_3_^−^ grown plants also accumulated a higher concentration of Mg^2+^ than NH_4_^+^-grown plants (Figure 7D). The highest concentration of NO_3_^−^ was found in NO_3_^−^-treated plants under DL; however, CL strongly reduced NO_3_^−^ concentration in the leaves, indicating a more efficient NO_3_^−^ assimilation under CL than under DL (Figure 7E). NH_4_^+^ grown plants contained a higher concentration of phosphate than NO_3_^−^-grown plants with a greater difference between N treatments under DL (Figure 7F). The supply of NH_4_^+^ enhanced accumulation of SO_4_^−^ in the leaves under DL but did not have a significant impact under CL (Figure 7G). The NH_4_^+^ supply also enhanced the accumulation of Cl^−^ over that seen with NO_3_^−^ supply at both DL and CL (Figure 7H).

## 4. Discussion

A full factorial experiment with a variation of light (DL vs. CL) and N form (NO_3_^−^ vs. NH_4_^+^), applied at the early stage of plant development, showed that the combined application of CL and NH_4_^+^ synergetically inhibited plant growth and leaf area expansion and induced leaf chlorosis. When CL and NH_4_^+^ treatments were applied at later stages and were shorter, no synergetic effect of the combined application of CL and NH_4_^+^ was apparent on plant growth or the appearance of chlorosis; however, analysis of xylem sap from decapitated plants revealed a synergetic modulation of hormonal long-distance root-to-shoot transport. This synergetic effect was strongly expressed for the stress hormones JA and ACC. Xylem sap analysis of N-deficient plants under DL showed up-regulation of JA and SA, but this was not observed for NH_4_^+^-treated plants grown under the same light conditions. An overlapping pattern of upregulation of stress hormones induced by N deficiency or by the combined application of CL and NH_4_^+^ indicate a potential role for JA under several abiotic stresses, where the JA function under specific conditions will be fine-tuned by modulation of the concentrations of other hormones.

### 4.1. Synergetic Effect of Combined Application of CL and NH_4_^+^

The balance between light capture and CO_2_ fixation is important for preventing photo-oxidative damage in leaves. Several investigations have shown that CL-induced injury is related to photo-oxidative stress and the accumulation of reactive oxygen species (ROS) in leaf cells [5]. For plants grown on a nutrient solution containing NO_3_^−^, the absorbed light is used for both the assimilation of carbon and the assimilation of NO_3_^−^. In tomato plants, NO_3_^−^ assimilation occurs predominantly in leaf cells, in parallel with CO_2_ assimilation [51]. Balanced photosynthetic electron transport requires a tight control of proper ratio between NADPH and ATP. This ratio strongly depends on NO_3_^−^ assimilation in leaves because NO_3_^−^ assimilation requires 2.5-fold more electrons than CO_2_ reduction to triose phosphate (TP) and 3-fold less ATP [51]. Therefore, replacement of NO_3_^−^ by NH_4_^+^ as the N source in the nutrient solution will adversely shift the balance between NADPH and ATP and generate surplus reductant that will then form ROS and induce oxidative stress. Plants under CL already have increased levels of ROS [52]; therefore, replacement of NO_3_^−^ with NH_4_^+^ might further exacerbate oxidative stress. The plant cells’ anti-oxidative machinery will not be capable of deactivating the accumulated ROS and eliminating oxidative stress. Therefore, the synergetic effect is seen between CL and NH_4_^+^ in terms of inducing oxidative stress and inhibiting plant growth.

Interestingly, under mild stress, the combine application of CL and NH_4_^+^ did not induce injury symptoms and plant growth inhibition. Instead, it enhanced the accumulation of JA and ACC in the xylem sap, indicating that long-distance root-to-shoot transport of these hormones was more sensitive than plant growth to the interaction effect between CL and NH_4_^+^. This suggests a potential role for JA and ethylene in plant adaptation to the combined application of CL and NH_4_^+^ supply.

JA is a multifunctional hormone with involvements in plant growth and development, as well as in plant responses to biotic and abiotic stresses [53,54]. The role of JA in plant growth primarily involves the inhibition of root growth, leaf expansion, and hypocotyl elongation [54,55]. In plant development, JA suppresses seed germination, inhibits apical hook formation in Arabidopsis, promotes trichome formation, delays flowering, modulates gravitropism, controls stamen development, regulates embryo/seed development and induces leaf senescence (for review see [55]). The role of JA in biotic stress is to induce a plant disease response against necrotrophic and herbivorous insect feeding [56,57]. The promotion of plant adaptation to abiotic stress by JA extends to many other stresses, including salt, drought, heavy metals, heat, and UV-radiation (reviewed in [58]).

Although JA, as a plant stress hormone, usually inhibits plant growth when applied externally, under stress conditions, JA can have a positive effect on growth through its activation of antioxidant defense enzymes and promotion of the accumulation of various secondary compounds with antioxidant activity. This enhanced antioxidant activity triggered by JA results in ROS deactivation and at least a partial growth recovery. For example, in tomato, endogenous JA enhanced salt tolerance mainly by maintaining ROS homeostasis [59] and the induction of ROS-avoidance enzymes [60]. Taking into account that the synergetic inhibitory effect of the combination of CL and NH_4_^+^ might be related to the imbalance and overproduction of ROS, the increase in JA concentration in the xylem sap could stimulate antioxidant activity, including ROS-avoidance enzymes and chemical antioxidants, to diminish the negative effect of the combined stresses.

The growth–defense tradeoff is a balance of plant resource distribution that defines whether plant invests into growth or defense, depending on external and internal factors [61]. For a long time, the growth-defense tradeoff has been considered as a model that describes the competition for resources that shift metabolic pathways into growth or into defense modes. A recent analysis of the crosstalk between JA and phytochrome B signaling pathways showed that the growth–defense tradeoff is guided at the signaling level, rather than being directly regulated through assimilate competition between growth and defense responses. Indeed, a genotype that combines mutations in JA and in phytochrome B signaling showed a decoupling of the growth–defense tradeoff and allowed the simultaneous combination of plant growth and defense responses [62]. The key role of JA in the growth–defense tradeoff raises the question of how one hormone can be involved in plant adaptation under different environmental conditions, including various types of biotic and abiotic stresses. The key hypothesis explaining this multifunctional role of JA is that under specific stress conditions, JA crosstalks with other hormones; therefore, JA is a core component in the plant response to biotic and abiotic stresses [63].

In support of this hypothesis, the combined application of CL and NH_4_^+^ showed a synergetic effect on JA as well as on ACC, the precursor of ethylene. This parallel increase in both JA and ethylene has been reported previously for another stress: infection with necrotrophic pathogens [64]. Under this condition, ethylene acts synergistically with JA to regulate the expression of the ERF branch pathway, whereas it antagonizes the MYC branch, resulting in a prioritization of the immune signaling network toward the JA-dependent and ethylene-dependent defense signaling associated with resistance to necrotrophs (reviewed in [65]).

### 4.2. The Absence of Synergetic Effects of Combined CL and NH_4_^+^ Application for Soluble Carbohydrates and Mineral ion Accumulation in Leaves

Under mild stress, where the synergetic effect of combined application of CL and NH_4_^+^ on hormone composition in xylem sap became apparent (Figure 5), no synergetic effects were observed for accumulation of soluble carbohydrates or mineral ions (Figure 6 and Figure 7). This means that the regulation of hormone composition in xylem sap was more sensitive than the regulation of accumulation of soluble carbohydrates or mineral ions in response to the combined application of CL and NH_4_^+^.

The CL in our experiment caused an increase in the starch content in the leaves, in agreement with other observations of an enhancement effect of CL on leaf starch accumulation [66]. This starch accumulation has been suggested to be related to the downregulation of photosynthesis and the induction of leaf chlorosis [67]. Under our conditions, NH_4_^+^ enhanced accumulation of starch in leaves under CL, indicating that this trait could contribute to the plant growth inhibition observed in NH_4_^+^-grown plants.

NH_4_^+^ also can stimulate the accumulation of soluble carbohydrates in leaves [8]. In agreement with this funding, we observed that, under DL, NH_4_^+^ enhanced the accumulation of soluble carbohydrates (Figure 6E,G). However, the combination of CL and NH_4_^+^ reduced the concentration of soluble carbohydrates compared with DL and NH_4_^+^ supply, indicating an antagonistic effect between CL and NH_4_^+^.

The ion imbalance induced by a predominant NH_4_^+^ supply was expressed as an excess accumulation of NH_4_^+^, PO_4_^3−^ and SO_4_^2−^ (Figure 7A,F,G) and a decrease in K^+^, Ca^2+^, Mg^2+^ (Figure 7B–D) and is considered one potential reason for the observed growth reduction [6]. Several investigations have shown that plants are more sensitive to the accumulation of NH_4_^+^ in the shoot than in the roots [68]; thus, NH_4_^+^ concentration in the shoot can be a promising trait for estimating plant resistance to NH_4_^+^. NH_4_^+^ accumulation can be prevented by a sufficient supply of carbon (C) skeletons and activity of the enzymes involved in NH_4_^+^ assimilation, namely glutamine synthase/ glutamate synthase (GS/GOGAT) reactions [69]. Normally, NH_4_^+^ assimilation occurs primarily in roots; however, a significant NH_4_^+^ concentration can be found in xylem sap when plants are grown under high NH_4_^+^ concentration [68]. The absence of any synergetic effect between CL and NH_4_^+^ precludes interaction with the regulation of ion concentration as being responsible for the synergetic effect observed for plant growth under strong stress. By contrast, for ammonium, accumulation of which in leaves could induce toxic effects, we found an antagonistic effect, which theoretically should diminish the effect of the combined application of CL and NH_4_^+^. Indeed, under CL, the level of NH_4_^+^ in the leaves strongly decreased (Figure 7A), which might reflect to the higher availability of C-skeletons delivered to the roots and used for NH_4_^+^ assimilation [6], which is indirectly supported by higher accumulation of starch, as the main reserve carbohydrate, in leaves for CL-grown plants (Figure 6H).

### 4.3. Plant Adaptation to N-Deficiency

LWR reflects the dry matter allocation between leaves and roots and is very sensitive to the level of N supply. In particular, N deficiency increases dry matter allocation to roots and contributes to the enhancement of the plant capacity for N acquisition [70]. Consistent with this, more DM was allocated to roots by N-deprived plants than by plants grown under non-limited NO_3_^−^ supply (Figure 3F).

One proposed hypothesis to explain the effect of NO_3_^−^ on dry matter allocation is that NO_3_^−^ itself plays a role as a signal [71]. This hypothesis was based on an experiment with NO_3_^−^ reductase tobacco mutants and knock-down tobacco lines that showed a close correlation between NO_3_^−^ concentration in the shoot and the shoot/root ratio. However, this hypothesis cannot explain our results in plants grown with NH_4_^+^ as the sole N source, where the dry matter allocation to the roots was the same as for plants grown with NO_3_^−^ as the sole N source (Figure 3F), as this indicated that dry matter allocation was independent of nitrate. Other studies support our data that N supplied exclusively as NH_4_^+^ (i.e., no NO_3_^−^ in solution) does not change the DM allocation to the roots [72].

An alternative hypothesis explains the effect of N deficiency on dry matter allocation between shoot and roots through a key role of protein concentration in the leaves [73]. In agreement with this hypothesis, in the present study, we did not expect to observe differences in dry matter allocation between NH_4_^+^-grown and NO_3_^−^-grown plants because NH_4_^+^ supply reportedly has no effect on the accumulation of total N and soluble proteins in shoots [72]. The presence of a correlation between soluble protein concentrations in shoots and dry matter allocation does not explain the molecular mechanism of this dependency. The simplest explanation is that a proportion of the N utilization occurring in roots depends on the N availability: a low N availability enhances N utilization in the roots and increases the competition between roots and shoots in favor of the roots [73]. However, we cannot exclude the possibility that hormonal signaling contributes to the regulation of resource allocation.

Nitrogen-deficient plants have higher RWR; however, no differences in LWR were observed (Figure 3C) because of the smaller DM investment in the stem. Therefore, the reduction in growth under N limitation cannot be explained by a decreased DM allocation to the leaves. However, the reduction in SLA by 48% under N deficiency (Figure 3D) indicates that this trait significantly contributes to plant growth reduction. The observation that specific morphological traits, such as DM allocation to roots (Figure 3F) and SLA (Figure 3D), were modulated under N limitation but were independent of N form indicates that the regulation of these traits was independent of signaling pathways related to specific N forms but remained sensitive to the internal N status of the plants. This assumes that different hormonal signaling pathways can be involved in plant growth responses to N deprivation and to different N forms.

The hormone ABA is synthesized in the roots in response to diverse stimuli (especially drought) and is transported to the shoots where it triggers stomatal closure and other physiological responses [74]. In our experiment, the increase in ABA concentration (Figure 4A) agrees with the finding of other ABA measurements in xylem sap obtained by exudation under root pressure [75,76]. However, when xylem sap was collected by pressurization of detopped root systems, no effect of N deprivation was found on the ABA concentration in the xylem sap [77,78]. Therefore, the increase in ABA concentration might be related to the method of collection, as explained by Dodd et al. [79]. N deprivation decreases the exudation rate from detopped root systems [80,81], while ABA concentration increases exponentially as the flux rate from the roots decreases [82,83]. Thus, sap exudate from detopped roots of N-deficient plants might be expected to show high ABA concentrations. Indeed, in our experiment, N deficiency strongly decreased xylem sap flux (by 80% in comparison with NO_3_^−^-fed plants) (Figure 3H); consequently, a low rate of xylem sap flux might contribute to higher concentrations of ABA.

Although the concentration of ABA increased twofold, the level of PA, which is a downstream metabolite of ABA, increased sevenfold, indicating that the utilization rate was higher for ABA than for PA. PA is considered a physiologically inactive form of ABA [46], so we did not expect to see a specific physiological response of plants to increases in PA concentration in xylem sap (Figure 4B).

Hormones other than ABA, such as SA, JA, and ethylene, are considered stress hormones and play significant roles in plant adaptation to biotic and abiotic stresses [84]. Our finding of strong increases in SA and JA concentrations in xylem sap (Figure 4E and Figure 5A,B) suggests that these hormones contribute to plant adaptation under N-limiting conditions. The increases in JA and SA concentrations under N deprivation support a role for these hormones in plant responses to the multiple stresses that plants often experience in nature. Previous investigations have shown that N deficiency could cause SA accumulation in Arabidopsis leaves [85] and enhance wound-induced JA levels in maize leaves [86]. Transcriptomic data showed that N deficiency could enhance both SA and JA signaling pathways [87]. However, in the roots of sorghum, N deficiency reduced SA and did not change JA concentrations [88], indicating that the regulation of SA and JA biosynthesis in response to low N supply can be species specific and may also depend on other environmental factors. Interestingly, the plant phenotype induced by low N might resemble the phenotypes induced by SA and/or JA. This phenotype is characterized by growth reduction and increased formation of lignin [89,90], as well as an accumulation of secondary defense metabolites [91] that are produced to reduce the incidence of disease and insect attack.

## 5. Conclusions

The sessile lifestyle of plants and the resultant unavoidable exposure to single and multiple environmental stresses would be expected to promote the evolution of mechanisms that allow plant growth adjustments in response to the surrounding environmental conditions. The presence of several plant hormones and the numerous possible combinations and concentrations of these hormones in plants suggest that specific hormone compositions in plants help in the adaptation to specific stress conditions. In agreement with this suggestion, the different, but overlapping, patterns of hormones responded to different types of stresses, such as N-deficiency or the combined application of CL and NH_4_^+^. JA was an overlapping regulatory hormone that was upregulated by both light and N stress. Given the well-known role of JA in growth, development, and plant responses to biotic and abiotic stresses, the findings presented here support a core role for JA in plant adaptation to various environmental stresses, including those imposed by adverse conditions of light and N supply. Further investigation resolving crosstalk between JA and other hormones will provide novel insights into and a better understanding of plant adaptation to abiotic stresses occurring singly or concomitantly with others.

## Data Availability

Data used is within the article or Supplementary Materials.

## Supplementary Materials

The following are available online at https://www.mdpi.com/2223-7747/10/3/573/s1, Figure S1: Images of plants from the experiment with strong stress shortly before sampling. Seedlings of both genotypes (cv. Ailsa Craig and cv. Rio Grande) were transferred at day 12 after sowing (DAS) to nutrient solution containing KNO_3_ or (NH4)_2_SO_4_ and grown under diurnal light. At DAS 15, one half of plants were maintained in diurnal light and the other half was transferred to continuous light. Plants were sampled at 26 DAS and 27 DAS for the continuous and diurnal light, respectively. Every treatment is represented by 4 plants. Table S1: Experiment with strong stress. Analysis of variance significance levels. Table S2: Experiment with mild stress. Analysis of variance significance levels. Table S3: Experiment with mild stress. Analysis of variance significance levels. Table S4: Experiment with mild stress. Analysis of variance significance levels. Table S5: Experiment with mild stress. Analysis of variance significance levels. Table S6: Experiment with mild stress. Analysis of variance significance levels.
