# Supplementary material for: Nitrogen Deficiency and Synergism between Continuous Light and Root Ammonium Supply Modulate Distinct but Overlapping Patterns of Phytohormone Composition in Xylem Sap of Tomato Plants
Source: Plants (Basel). 2021 Mar 18;10(3):573. doi: 10.3390/plants10030573 (PMC8003008; doi:10.3390/plants10030573)
Supplement: Supplementary file 1 [file plants-10-00573-s001.zip › Tables_ALL04.pdf]

Table S1. Experiment with strong stress. Analysis of variance significance levels. Variables are leaf area, plant biomass, leaf weight ratio (LWR), specific leaf area (SLA), leaf dry matter content (LDM), chlorophyll index. The data was analyzed by 3-factor-ANOVA, using factors Genotype (cv. Ailsa Craig or cv. Rio Grande), N from (NH_4^+ or NO_3^-), and light (diurnal or continuous). Means and SE are presented in Figure 1. If the effect of a factor or the interaction between factors was significant, we labeled it with asterisks, * p<0.05, ** p<0.01, *** p<0.001. NS is nonsignificant at the 0.05 probability level.

Source of variation	Leaf area (cm^2)	Plant biomass, (g)	LWR (%)	SLA (cm^2 g^{-1})	LDM (%)	Chlorophyll index
Genotype (G)	**	***	NS	NS	NS	**
N form (N)	***	***	***	***	***	***
Light (L)	NS	NS	***	NS	***	***
G × N	**	**	NS	NS	NS	NS
G × L	NS	NS	*	NS	NS	**
N × L	*	***	NS	***	NS	***
G × N × L	NS	NS	NS	NS	NS	*

Table S2. Experiment with mild stress. Analysis of variance significance levels. Variables are leaf area, plant biomass, leaf weight ratio (LWR), specific leaf area (SLA), leaf dry matter content (LDM), root weight ratio (RWR), root dry matter content (RDM), and xylem sap rate per h. The data was analyzed by 2-factor-ANOVA, using NH_4^+ at 2 levels and NO_3^- at two levels. Interaction between N form and light periodicity was analyzed by 2-factor ANOVA, using N from (NH_4^+ or NO_3^-) and light (diurnal or continuous). Means and SE are presented in Figure 3. If the effect of a factor or the interaction between factors was significant, we labeled it with asterisks, * p<0.05, ** p<0.01, *** p<0.001. NS is nonsignificant at the 0.05 probability level.

Source of variation	Leaf area (cm^2)	Plant biomass, (g)	LWR (%)	SLA (cm^2 g^{-1})
NH_4^+	*	NS	*	*
NO_3^-	***	***	NS	**
$NH_4^+ \times NO_3^-$	***	*	**	**
N form (N)	***	***	NS	NS
Light (L)	***	***	***	***
N x L	NS	**	NS	NS
	LDM (%)	RWR (%)	RDM (%)	Xylem sap (ml $plant^{-1}$ h^{-1})
NH_4^+	***	***	**	***
NO_3^-	***	***	**	***
$NH_4^+ \times NO_3^-$	NS	***	***	NS
N form (N)	NS	NS	NS	***
Light (L)	***	***	NS	*
N x L	**	NS	NS	**

Table S3. Experiment with mild stress. Analysis of variance significance levels. Variables are ABA, PA, IAA, OxIAA,, SA, BzA concentrations. The data was analyzed by 2-factor-ANOVA, using NH_4^+ at 2 levels and NO_3^- at two levels. Interaction between N form and light periodicity was analyzed by 2-factor ANOVA, using N from (NH_4^+ or NO_3^-) and light (diurnal or continuous). Means and SE are presented in Figure 4. If the effect of a factor or the interaction between factors was significant, we labeled it with asterisks, * p<0.05, ** p<0.01, *** p<0.001. NS is nonsignificant at the 0.05 probability level.

Source of variation	ABA	PA	IAA	OxIAA	SA	BzA
NH_4^+	NS	**	NS	NS	**	NS
NO_3^-	*	**	*	NS	*	NS
$NH_4^+ \times NO_3^-$	NS	**	NS	*	***	NS
N form (N)	NS	NS	NS	NS	NS	NS
Light (L)	NS	*	NS	NS	NS	**
N x L	NS	NS	NS	NS	*	NS

Table S4. Experiment with mild stress. Analysis of variance significance levels. Variables are JA, JA-Ileu, tZR, cZR, iP7G, ACC concentrations. The data was analyzed by 2-factor-ANOVA, using NH_4^+ at 2 levels and NO_3^- at two levels. Interaction between N form and light periodicity was analyzed by 2-factor ANOVA, using N from (NH_4^+ or NO_3^-) and light (diurnal or continuous). Means and SE are presented in Figure 5. If the effect of a factor or the interaction between factors was significant, we labeled it with asterisks, * p<0.05, ** p<0.01, *** p<0.001. NS is nonsignificant at the 0.05 probability level.

Source of variation	JA	JA-Ileu	tZR	cZR	iP7G	ACC
NH_4^+	*	NS	*	NS	NS	NS
NO_3^-	*	NS	NS	NS	NS	NS
$NH_4^+ \times NO_3^-$	NS	*	NS	NS	NS	NS
N form (N)	*	*	*	NS	NS	**
Light (L)	NS	**	**	*	***	**
N x L	*	**	*	NS	*	**

Table S5. Experiment with mild stress. Analysis of variance significance levels. Variables are total nitrogen (N), total chlorophyll, chlorophyll a/b ratio, total carotenoids, glucose, fructose, sucrose, starch. The data was analyzed by 2-factor-ANOVA, using N form (NH_4^+ or NO_3^-) and light (diurnal or continuous) as factors. Means and SE are presented in Figure 6. If the effect of a factor or the interaction between factors was significant, we labeled it with asterisks, * p<0.05, ** p<0.01, *** p<0.001. NS is nonsignificant at the 0.05 probability level.

Source of variation	Total N (mg g^{-1})	ChlA+ChlB, mg/g	Chl A/B ratio	Carotinoids mg g^{-1} DW
N form (N)	NS	NS	NS	NS
Light (L)	***	**	***	***
N x L	NS	NS	NS	NS

	Gluc (mg g^{-1} DW)	Fruc (mg g^{-1} DW)	Suc (mg g^{-1} DW)	Starch (mg g^{-1} DW)
N form (N)	***	***	**	*
Light (L)	*	NS	NS	***
N x L	*	*	NS	NS

Table S6. Experiment with mild stress. Analysis of variance significance levels. Variables are concentrations of NH_4^+, K^+, Ca^{+2}, Mg^{+2}, NO_3^-, $H_2PO_4^-$, SO_4^{-2}, Cl^-. The data was analyzed by 2-factor-ANOVA, using two N forms (NH_4^+ or NO_3^-) and two light conditions (diurnal or continuous) as factors. Means and SE are presented in Figure 7. If the effect of a factor or the interaction between factors was significant, we labeled it with asterisks, * p<0.05, ** p<0.01, *** p<0.001. NS is nonsignificant at the 0.05 probability level.

Source of variation	NH_4^+ (µg g^{-1} DW)	K^+ (mg/g DW)	Ca^{+2} (mg g^{-1} DW)	Mg^{2+} (mg g^{-1} DW)
N form (N)	***	***	***	***
Light (L)	***	NS	*	NS
N x L	***	NS	NS	NS

	NO_3^- (mg/g DW)	$H_2PO_4^-$ (mg/g DW)	SO_4^{-2} (mg/g DW)	Cl^- (µg g^{-1} DW)
N form (N)	***	***	*	***
Light (L)	***	NS	NS	NS
N x L	***	**	NS	NS
