# Supplementary figures and images for: Nitrogen Deficiency and Synergism between Continuous Light and Root Ammonium Supply Modulate Distinct but Overlapping Patterns of Phytohormone Composition in Xylem Sap of Tomato Plants
Source: Plants (Basel). 2021 Mar 18;10(3):573. doi: 10.3390/plants10030573 (PMC8003008; doi:10.3390/plants10030573)

## Diurnal Light

$\text{NO}_3^-$

$\text{NH}_4^+$

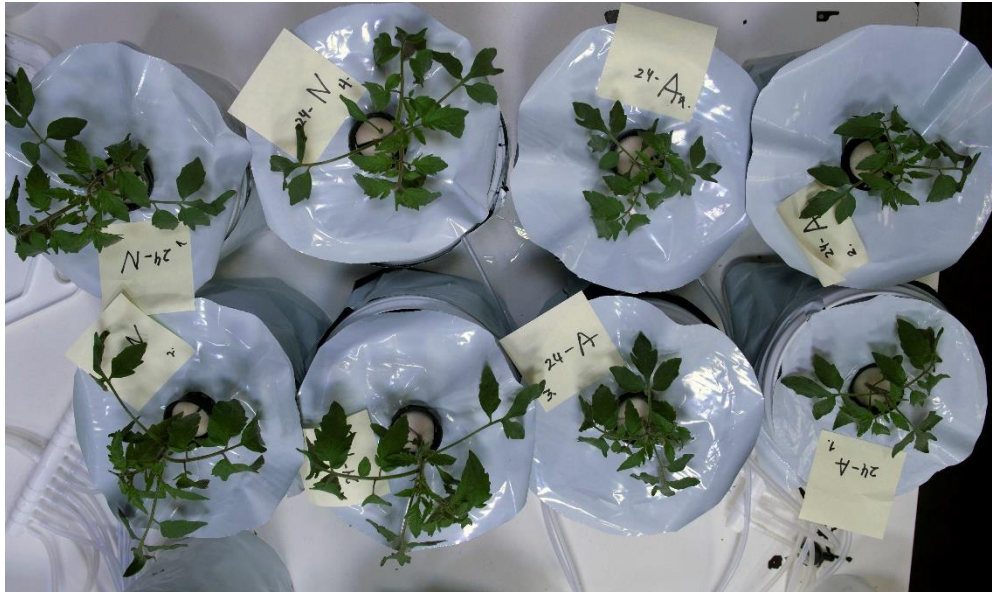

Ailsa Craig

## Continuous Light

$\text{NO}_3^-$

$\text{NH}_4^+$

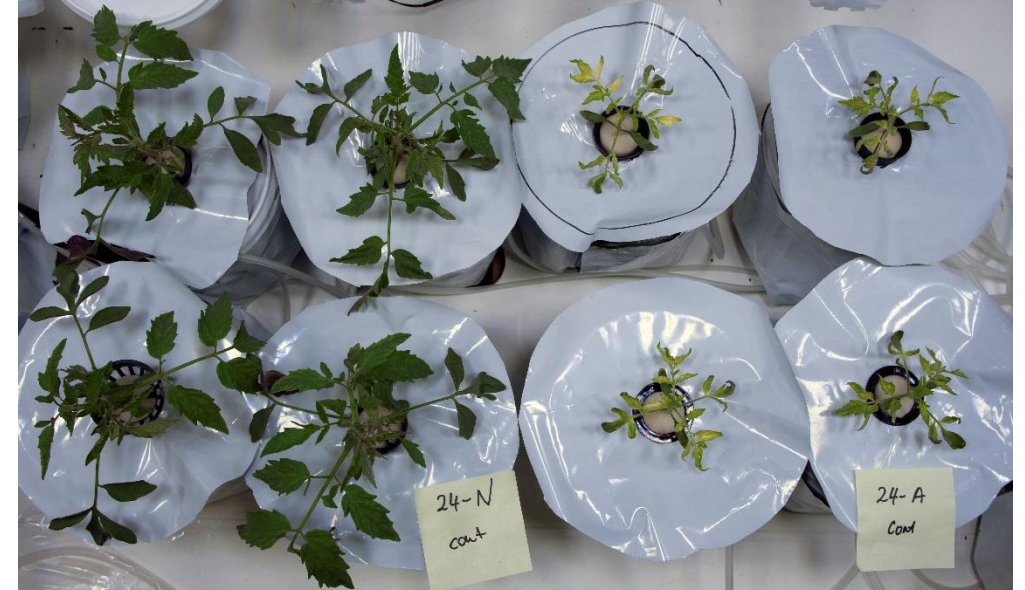

Rio Grande

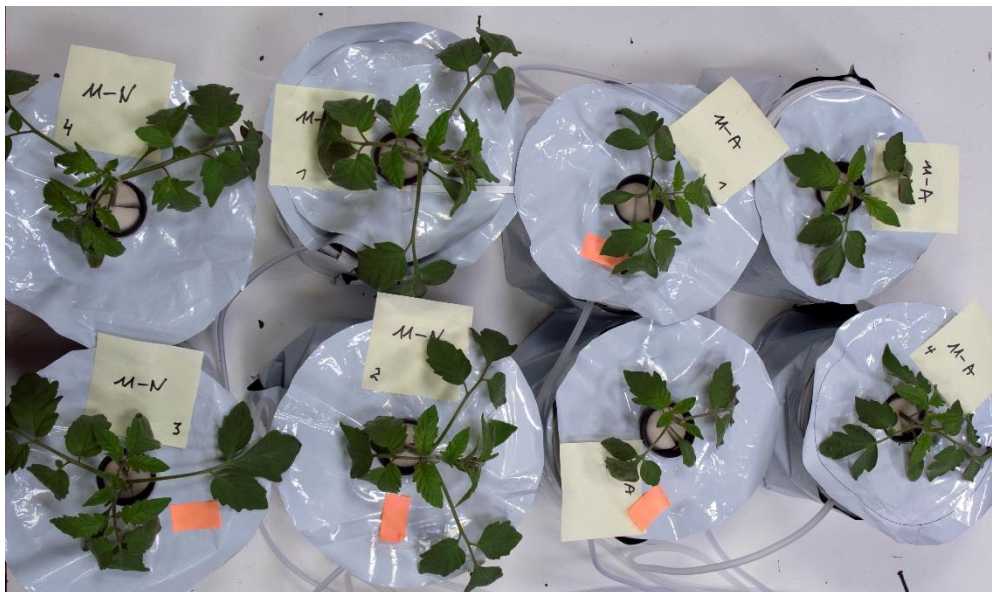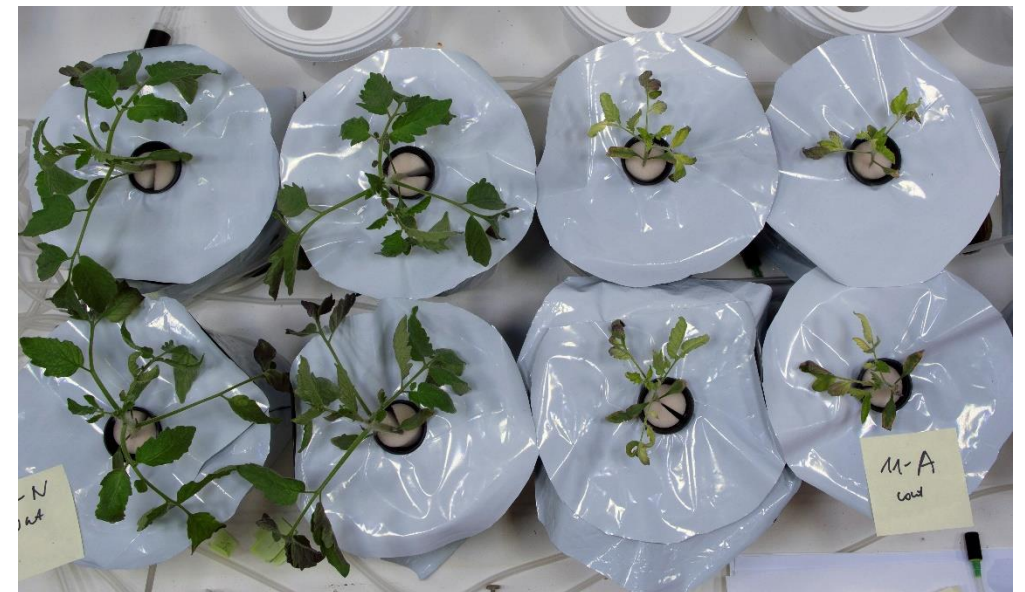

Supplement: Supplementary file 1 [file plants-10-00573-s001.zip › SupplFig01.pdf]
